# Ambition With Uncertainty: Exploring Policy-Makers’ Perspectives on Pathways to Net Zero Healthcare

**DOI:** 10.34172/ijhpm.8440

**Published:** 2025-01-15

**Authors:** Anand Bhopal, Kristine Bærøe, Ole F. Norheim

**Affiliations:** ^1^Bergen Centre for Ethics and Priority Setting, Department of Global Public Health and Primary Care, Faculty of Medicine, University of Bergen, Bergen, Norway.; ^2^Centre for Energy and Climate Transformation (CET), University of Bergen, Bergen, Norway.; ^3^Takemi Program in International Health, Department of Global Health and Population, Harvard T.H. Chan School of Public Health, Harvard University, Boston, MA, USA.; ^4^Department of Global Public Health and Primary Care, Faculty of Medicine, University of Bergen, Bergen, Norway.; ^5^Department of Global Health and Population, Harvard T.H. Chan School of Public Health, Harvard University, Boston, MA, USA.

**Keywords:** Climate Change, Net Zero, Priority Setting, Trade-offs, Sustainable Healthcare

## Abstract

**Background:**

Over 80 countries have now signed up to the COP26 Health Programme—a World Health Organization (WHO)-led initiative on climate change and health—of which 45 countries have committed to reaching *net zero* emissions before 2050. Efforts to reduce healthcare’s carbon footprint raise conceptual, ethical and practical challenges for efficient and fair resource allocation. This study investigates how civil servants leading the development and implementation of national net zero healthcare strategies conceptualise the responsibility of health systems to cut emissions and describe potential trade-offs along the way.

**Methods:**

We undertook 11 online, semi-structured qualitative research interviews between September 2022 – May 2023 with civil servants leading national net zero healthcare strategies. The interview guide explored three main areas: responsibility for emissions, priority setting and international perspectives. Interviews were coded and analysed the data using Malterud’s systematic text condensation (STC).

**Results:**

Four main themes emerged: obligation to act, leadership, governance, and prioritization. Participants described that the healthcare system should take responsibility for its entire carbon footprint, including harms inflicted beyond national borders. We also found indications of synergistic, multi-scalar health leadership—clinical, civil service, and political—helping to accelerate the net zero healthcare agenda. Participants generally rejected the notion of direct "trade-offs" between efforts to reduce emissions and patient care, emphasising ways net zero healthcare can leverage societal health improvements more broadly. These empirical findings inform the emerging literature exploring how health systems should account for their environmental impacts.

**Conclusion:**

Our findings highlight the sincerity of ambitions to deliver net zero healthcare and uncertainties on how to get there. Further work characterising the types of constraints and trade-offs policy-makers face on the path to net zero healthcare systems, including examples of how these have been overcome, could help integrate climate concerns into healthcare decision-making and resource allocation processes.

## Background

**Key Messages**

**Implications for policy makers**

- It is increasingly clear that healthcare is not only at risk from climate change but also a large polluter and should do its part to mitigate its climate impact.
- Across the world, countries are developing net zero emissions healthcare systems, spearheaded by the World Health Organization’s (WHO’s) initiative for sustainable, low-carbon, climate resilient health systems.
- Net zero commitments raise new challenges for fair resource allocation in healthcare, which has not traditionally incorporated climate impacts, raising potential trade-offs for health system leaders which should be met on.
- This research highlights the sincerity of ambitions to deliver net zero healthcare and uncertainties on how to get there in ways which best protect and improve health.
- Developing a richer empirical understanding of the constraints and trade-offs facing civil servants and policy-makers is crucial as net zero healthcare rapidly shifts from awareness raising to implementation.

**Implications for the public**

Climate change poses a present and growing threat to health. Keeping global temperature to below 1.5-2 °C in line with the Paris Agreement requires rapidly reducing global greenhouse gas emissions. Over the last decade, it has become increasingly clear that the health sector is not only at risk from climate change, but also a significant polluter and should do its part to cut emissions. A global movement is now underway to decarbonize healthcare systems. Over 80 countries have now signed up to the World Health Organization (WHO) programme for sustainable, low-carbon healthcare systems, with 45 countries committing to developing a *net zero healthcare system *by 2050. The initiative for net zero healthcare systems poses new challenges and opportunities for health systems which have traditionally not taken its carbon footprint into account. This study explores how civil servants leading this work conceptualise and navigate any potential trade-offs on the pathway to net zero healthcare.

In 2021, the World Health Organization (WHO) and partners launched the COP26 Health Programme for climate resilient and low-carbon health systems. To date, over 80 countries have since signed up, of which 45 countries have committed to developing a net zero emissions health system before 2050^1^ (Box 1). This marks the first time a dedicated Health Programme has been promoted at the annual United Nations Conference of Parties (COP) climate negotiations. Its success reflects the growing international consensus regarding the vulnerabilities of the health sector to climate change, as well as an awareness that healthcare systems, especially in wealthy countries, can and should do more to reduce their carbon footprint. WHO has since launched the Alliance for Transformative Action on Climate and Health (ATACH) to help turn these political commitments into action. This platform supports countries with a combination of advocacy, technical assistance, knowledge sharing, monitoring, and financing.^2^ The inaugural “Health Day” and climate-health inter-Ministerial meeting at COP28,^3^ as well as the resulting *COP28 Declaration on Climate and Health* endorsed by over 120 countries,^4^ highlights widening interest in the health sector’s role in responding to climate change. For national policy-makers tasked with delivering the net zero healthcare commitments, cutting healthcare emissions in the face of other challenges—eg, limited financial budgets, the fallout of the COVID-19 pandemic, and ageing populations—presents a range of practical, ethical and conceptual challenges and trade-offs for healthcare resource allocation.^5,6^ With net zero healthcare rapidly shifting from conceptualization to implementation it is vital to understand which actions are being taken and why.^7-9^

**Box 1. Defining Net Zero and Net Zero Healthcare**

“Net Zero” is a concept enshrined in the Paris Agreement, the near-universally ratified, legally binding treaty to limit global warming, and the second major treaty to emerge from the UNFCCC.^18^ Unlike its predecessor, the Kyoto Protocol, which limited emission reduction commitments to a sub-set of industrialized countries, the Paris Agreement encourages multi-scalar climate leadership by facilitating pledges from all countries, as well as non-state and sub-national actors^19^ – including the healthcare sector.^20^ Achieving net zero requires a combination of *reducing* emissions, through phasing out fossil fuel use, and *removing* emissions, through planting trees or capturing carbon dioxide from the air and storing it underground; this reflects the *net* in net zero. To meet the Paris Agreement, global policy targets have coalesced around the target of reaching net zero by 2050, with sectoral targets.^21^

As signatories to the COP26 Health Programme, countries are required to undertake baseline emissions assessments, develop an action plan or roadmap for a sustainable low-carbon healthcare system, and ideally commit to a date to achieve net zero healthcare before 2050. In a recent review, Hough and Tanugi-Carresse^22^ found a growing number of countries have undertaken baseline emissions assessments but still only a few detailed national net zero healthcare plans. The clearest example of a net zero healthcare roadmap is the English strategy for “Delivering a net zero National Health Service”^23^ which has now been embedded into legislation.^24^ This NHS England net zero strategy also discusses how to address residual emissions (ie, emissions remaining after all identified interventions have been delivered) through research, innovation and potentially offsetting mechanisms.

---------------

Abbreviations: UNFCCC, United Nations Framework Convention on Climate Change; NHS, National Health Service.

Healthcare’s carbon footprint currently accounts for between 4%-5% of global emissions with large variation within and between countries and regions.^10^ Emissions are far higher in high-income countries while the health impacts of climate change are concentrated among low- and middle-income countries (LMICs),^11^ highlighting the centrality of equity and fairness to healthcare decarbonization. There is also considerable variation between high-income countries; for example, per-capita emissions vary more than threefold between the United States (1.7 tonnes) and England (0.54 tonnes) without commensurate benefits in healthcare access, coverage and quality.^12,13^ Pichler et al^14^ have identified three factors which drive over half of the variation between countries: carbon intensity of the domestic energy system, the energy intensity of the domestic economy and national healthcare expenditure. This highlights the mitigation potential from interventions outside of the health sector, as well as the need for direct actions from within the health sector itself.^5^

 Healthcare’s carbon footprint spans the entire system, from the production and procurement of goods, often overseas, to clinical practices closer to home. To avoid double counting of mitigation efforts, emissions are commonly separated into different “Scopes” following the Greenhouse Gas Protocol^15^: Scope 1 (direct emissions incurred on site), Scope 2 (emissions from purchased energy), and Scope 3 emissions (all other emissions embedded in the supply chain). The scopes of emissions which are included in estimates are important since the majority of healthcare’s carbon footprint lies in the supply chain.^12,13,16,17^ In England, for example, the main contributors to the health sector’s carbon footprint are patient and staff travel (10%), anaesthetic gases and inhalers (5%), building energy (10%), water and waste management (5%), and the (Scope 3) supply chain of health services (62%), which includes pharmaceuticals and chemicals (20%) and medical equipment (10%).

 To date, the qualitative literature examining the emerging net zero healthcare agenda has primarily focused on low-carbon clinical practices, including health professionals’ perspectives on how to decarbonize medical specialties^25,26^ and shift to more sustainable prescribing approaches.^27,28^ Some work has integrated the perspectives of clinicians and administrative staff in order to better understand potential mitigation strategies and challenges to implementation,^29-32^ as well as motivators to engage in sustainability initiatives and advocacy.^33^ Two studies have incorporated participants’ perceptions of the responsibility of the healthcare sector to decarbonise. Quittmann et al^34,35^ examine clinical and administrative perspectives within a large hospital in Germany, finding that while climate change in general is seen to be important, stakeholders did not feel cutting emissions was a priority. Instead participants perceived a trade-off with patient care and identified multiple organizational and structural barriers to implementation. Fylan and Allison^29^ explored health professionals, patients and citizens perspectives in northeast England through a series of deliberative workshops. They found a “rhetoric-reality gap” in which sustainability concerns widespread but directed away from individual agency towards organisational factors, at which level it is not prioritised. They also find evidence of a “moral offset” amongst participants, whereby the task of saving lives is considered to exempt the health service from reducing emissions.

 A critical stakeholder group overlooked from the net zero healthcare literature to date is policy-makers, ie, individuals involved in creating and implementing government policies.^36-38^ In this study we focus on civil servants, individuals helping to both conceive policy alternatives and execute political decisions.^39^ At this early stage in the net zero healthcare agenda, civil servants play a key role in conceptualizing the problem, determining the relevance to the health sector, and communicating with different audiences. This includes informing the political decision, taken by a country’s Minister of Health, to sign up to the COP26 Health Programme, and determining how to deliver upon these commitments.^40^

 Participants in this study are all leading the development and implementation of a national net zero healthcare strategy, with the exception for one participant from a prominent international health agency working on climate change and health (Participant 3). The countries represented are all members of the ATACH, which provides participating individuals an international platform to exchange views, share information, and enhance technical and political co-operation.^41^ Eight participants represent high-income countries. We incorporate two LMICs (Participants 5 and 8) as counterbalancing perspectives in the analysis.

 The net zero healthcare agenda remains in its infancy, with ongoing uncertainties on what should be done and at what costs.^8,10,42^ This study investigates how civil servants leading the development and implementation of national net zero healthcare strategies conceptualise the responsibility of health systems to cut emissions and any potential trade-offs along the way. Our aim is to develop a richer empirical understanding of constraints and trade-offs facing civil servants and policy-makers, helping to align research and policy agendas as net zero healthcare rapidly shifts from awareness raising to implementation.

## Methods

###  Research Design

 We conducted a series of semi-structured qualitative interviews with civil servants leading the development and implementation of national net zero healthcare strategies. Individuals were located within the respective country’s Ministry of Health, Directorate of Health or Public Health Agency. The study has been conducted as part of broader work examining the ethical implications of the net zero healthcare agenda.

###  Sampling and Recruitment

 We used a purposive sampling strategy to identify participants, including countries which had signed up to the COP26 health program and set a specific date to achieve net zero healthcare. Recruitment took place between July 2022 – April 2023. There were initially 8 email invitations (contact details were obtained from the website of the government department) with snowball sampling. In July 2022, the WHO Climate Change and Health Unit shared an invitation letter on our behalf with regional and country contacts to the ATACH. This did not result in any responses. We also placed two emails on a mailing list of environmentally engaged clinicians (“Doctors for Sustainable Healthcare” and Healthcare Without Harm), in December 2022 and March 2023, which yielded four additional country contacts. Overall, we identified and contacted relevant persons in 18 countries (11 high-income, 7 low- and middle-income) and 4 international organisations (Table).

**Table:** Participant Characteristics

Characteristics	
Gender (% female)	22
Age (y)	Under 45 (4), Over 45 (7)
Regional representation (by WHO world region)	Africa (1), Americas (2), Eastern Mediterranean (0), Europe (7), South-East Asia (1), Western Pacific (0)
Country income group (by World Bank classification)	Low-income (1), lower-middle income (0), upper-middle income (1), high-income (8)
Organisational representation (by Government agency)	Ministry of Health (5), Health Services Directorate (4), Public Health Directorate (1)

Abbreviation: WHO, World Health Organization.

###  Data Collection

 In total, 11 semi-structured qualitative research interviews were undertaken between September 2022 – May 2023; involving 8 high-income countries, 2 LMICs and 1 representative from a prominent international health agency working on climate change and health. All interviews were undertaken via video link (using *Microsoft Teams* or *Zoom* software). The interview guide (available in Supplementary file 1) followed three main themes: responsibility for emissions, priority setting and international perspectives. Interviews were audio recorded, transcribed verbatim and pseudonymized with unique identifiers on a password protected laptop owned by the University of Bergen, and safely stored on the encrypted *Microsoft One Drive* server.

 Three authors are involved in this study. The first author led the study, including data collection, analysis, and manuscript writing. They are a medical doctor with a longstanding interest in climate change and public health, particularly the ethical and policy dimensions of net zero healthcare. This has influenced the design of the interview guide and framing of the net zero challenge around the United Nations Framework Convention on Climate Change (UNFCCC) principles. The second author is a philosopher of science working within the field of ethics and philosophy of science. The third author is a medical doctor and philosopher working on fair priority setting in health.

###  Data Analysis 

 Data were manually transcribed, then coded and analyzed following Malterud’s systematic text condensation (STC).^43^ All authors conceptualized the study and planned the analysis. Two authors (AB and KB) independently read the interviews in full and identified preliminary themes. AB undertook the analysis and wrote the manuscript. All authors gave regular feedback during the analysis, reviewed drafts and agreed the final manuscript. While the STC coding process is inductive, our thought process throughout this study—from the aims of the study to the development of the interview guide and the coding process—has been informed by the guiding principles of the UNFCCC^44^ and the ethics work of the International Panel on Climate Change.^45^

## Results

 As both a concept and a practice, net zero healthcare is still in its early phases. During the interviews, the need for urgent action in the face of uncertainty and policy constraints came to the fore. From our analysis we identified four main themes (Figure): (*i*) Obligations to act (“*Unravelling obligations to act”*), (*ii*) leadership (“*Multi-scalar leadership”*), (*iii*) governance (“*Orchestrating ambitiousness”*), and (*iv*) prioritization (“*Prioritising net zero*”). *Unravelling obligations to act*, describes the intersecting responsibilities at the level of individual clinicians, health systems and global justice. *Multi-scalar leadership, *discusses the policy dimensions of net zero healthcare with a focus on civil servants. *Orchestrating ambitiousness*, describes the wider policy context, including new potential avenues for international collaboration across healthcare systems. *Prioritising net zero*, explores how participants conceptually balance net zero with other health sector goals.

**Figure:**
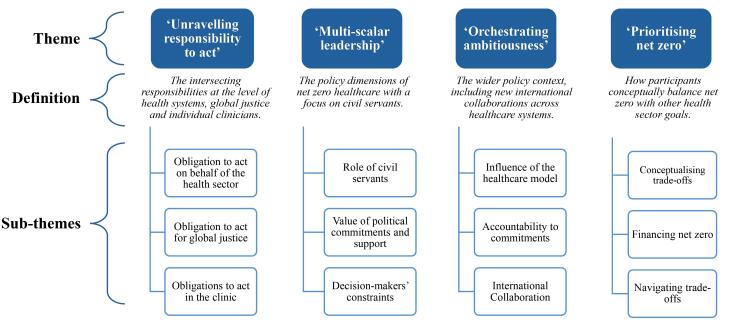
Overview of the Analysis Themes and Sub-themes.

###  Unravelling Obligations to Act

 Within the *Obligations to act* theme there are three sub-themes: “*Obligation to act on behalf of the health sector,” *describing views on the health sector’s responsibility to cut emissions; *“Obligation to act for global justice,”* exploring how this responsibility could and should be shared globally; and “*Obligations to act in the clinic*,” discussing the role of healthcare professionals in the net zero healthcare agenda.

####  Obligation to Act on Behalf of the Health Sector

 The health sector was seen to have a responsibility to cut emissions, both as a public service and in keeping with the health sector’s commitment to avoid harm. This was broadly framed in terms of trust: the health sector is full of trusted professionals who are individually and collectively bestowed with a responsibility to show leadership on societal issues affecting health – including climate change. One participant described the failure to act on healthcare’s carbon footprint as contravening the health sector’s “social mission.”

 “*I don’t think there is anyone that is unsympathetic to the challenges health systems face – and to the real demands that they are up against – but as a society we all have to contribute to tackling climate change and the harm that it creates. I think for healthcare organisations that is especially aligned with the mission” *[P6].

 Participants from different income-groups focused on different dimensions of net zero healthcare. High-income country participants focused on how to cut emissions to meet net zero goals efficiently, in ways which protect and improve healthcare. Some participants emphasized that this isn’t about some abstract benefit of reducing carbon, but rather that pursuing net zero can and should directly improve patient care. The focus amongst LMIC participants was more directed towards the harms of climate change itself. As one of the participants put it, the health system is the main line of defence against climate change, therefore, building climate resilient, low-carbon health systems is a priority.

 Scope 3 supply chain emissions were considered equally important for the health system to tackle as Scope 1 and 2 emissions; participants reflected that Scope 3 emissions are, however, less directly under their control. As one participant put it: “*I don’t know if [different emission scopes] affects our responsibility, I think it impacts the way we address them” *[P11]. A dominant framing used by participants was agency: “*health systems have control over their decisions and ultimately have a 100% responsibility to act on 100% of emissions” *[P2]. This represents a widely shared view amongst participants that Scope 3 emissions, accounting for the majority of the healthcare carbon footprint, are too big to ignore.

####  Obligation to Act for Global Justice 

 Participants understood and described a rationale for taking a global perspective, including that climate change will worsen already wide health inequalities. All countries were seen to have a role in cutting emissions from the health sector; however, participants did not consider global responsibilities to be directly relevant to their work. As one participant put it: *“You don’t say these countries have to do something and these countries don’t. You say these countries have to do something and these countries can afford to take a little bit longer – but everyone has to run at this” *[P2].At the same time, participants acknowledged practical challenges to implementing net zero healthcare globally. Particularly, as one participant put it, since there are no globalmechanisms, net zero healthcare is, practically speaking, a national effort:

 “*Responsibility should be seen at the country level and then scaled down at the local, or even facility level. I will tell you why – because there are no obligatory mechanisms at the global level that can push or interfere” *[P3].

 While high-income country participants described a responsibility incumbent upon them, as historical polluters, to support poorer countries to deliver net zero healthcare, they felt constrained, practically, by structural barriers – namely that their work remit focuses on decarbonizing healthcare nationally. LMIC participants focused more on the responsibility upon high-income countries to honour their historical responsibilities by providing greater financial and technical support.

####  Obligations to Act in the Clinic 

 Participants described how clinicians had first-hand experience of the health impacts of climate change. The phrase “do no harm” was often used as a rationale for why healthcare professionals and the health sector should take action to reduce emissions. Expanding on this idea, one participant reflected how all economic activities cause emissions, which in turn cause harm: “*I don’t believe we can do no harm at all, but I think we should do less harm than we currently do” *[P7].Clinicians were felt to hold considerable autonomy over healthcare practices, guideline setting and decision-making which can be amended to account for climate impacts. Several participants described a corollary responsibility for clinicians, in their role as advocates, to speak out and take action to reduce emissions. Senior clinicians, especially those in government or leading professional organisations, were seen to have a particularly important role in shifting healthcare norms to incorporate climate concerns. As one participant put it, there is a certain “*civil responsibility” *upon clinicians in government who are entrusted with the task of providing scientific advice to take this issue seriously.

###  Multi-scalar Leadership 

 We identified three sub-themes within the leadership theme: “*Role of civil servants,”* exploring how participants engaged with net zero healthcare in their position in the policy process; “*Value of political commitments and support,”* describing the value they placed participants placed upon engagement from politicians and senior leaders; and “*Decision-makers constraints,”* discussing the perceived constraints of health leaders and the information required to support decision-making.

####  Role of Civil Servants

 The emergence of the net zero healthcare agenda was considered to be both rapid and widespread, despite relatively few resources and little political clout. The COP26 Health Programme was viewed to have played a central role, both by drawing attention to the issue and raising the profile of work often already underway to reduce the environmental impacts of healthcare. However, this work was often described as driven from the “bottom-up” through the dedication and passion of people working at different levels of the health sector. Multiple participants described how they personally helped advance the net zero healthcare agenda, including by bringing colleagues together from across the health system to explore this issue.

 “*The reality is that this wasn’t something that was top-down driven, it was something that was driven by myself, as a middle manager, essentially you know within Government, and colleagues with an interest who cared about this subject, within health boards” *[P4].

 The net zero agenda was seen to be popular amongst staff throughout the healthcare system. Developing a strategy with specific goals was viewed as a good way to show impact, including of work which had already been undertaken. Alongside awareness-raising, a recurring theme was the need to build up the capacity of staff to deliver the work.

 “*There is so much enthusiasm and I’m not sure we’ve totally figured out how to really harness that. We could probably be doing more and going faster if we could just figure out how to tap into or how to give more direction to people who want to be a part of the solution” *[P11].

 Participants framed the capacity building challenge not only around funding but also the challenges of recruiting a cadre of experts with the right expertise – especially clinicians who understand the whole system.

####  Value of Political Commitments and Support

 The COP26 health programme was considered to have played a central role in building national support for net zero healthcare. In particular, the way the collective goals and involvement of WHO lends the programme a global reach. As one participant reflected, although health representatives had always attended COP meetings, they had been on the fringes; the COP26 Health Programme helped bring the health sector into fray. Since the COP26 health programme is a political commitment, signed onto by government officials, participants described it as having an important support function.

 “*Making the COP26 Health Programme commitment was a way of further legitimising the importance of the topic and the work we do… and keep it on the agenda of the Ministry” *[P10].

 Net zero healthcare was viewed as an intersectoral agenda, requiring collaboration beyond international borders, across government ministries and between private and public actors. Participants described that a common net zero framework has made intersectoral work easier, with potential to leverage additional benefits for public health.

####  Decision-Makers’ Constraints

 Participants described how both the public and politicians are increasingly concerned about climate change and health, which is helping to sustain the momentum of the net zero healthcare agenda. However, for better or worse, health service leaders and Ministers get judged by metrics and in most countries this has not, until very recently, included net zero. Decision-makers were described as facing a series of constraints that make it difficult for them to prioritize this issue. This includes some ambiguity on the costs involved in achieving net zero healthcare, such as the extent of upfront investments.

 “*Some things will provide a return on investment, other things won’t – that’s the bit that we really need to get a better understanding of because that’s where our Ministers will have to make some choices” *[P1].

 In part, this was seen to be about control mechanisms and the core functions of a Ministry of Health, which has not traditionally included climate, as opposed to relatively “concrete” problems like hospital waiting times. As one participant put it, health leaders are often under fire and understandably reluctant to make “*grand gestures”* they cannot deliver upon.

###  Orchestrating Ambitiousness

 Within the *Governance* theme are three sub-themes: *“Influence of the healthcare model,” *in which participants broadly explored the nexus between the health policy context and net zero commitments; “*Accountability to commitments,”* describing the rationale for developing a net zero healthcare strategy and the perceived importance of ensuring it is credible; and “*International collaboration,”* discussing the potential opportunities and challenges of working across health systems.

####  Influence of the Healthcare Model 

 The structure of a country’s health system and the national policy context were seen to be central to understanding countries net zero commitments and their potential to deliver upon current targets. Pre-existing national net zero targets—either for the country as a whole or for the public sector—provided a hook for policy-makers to more easily commit to the COP26 health programme. Making national commitments was perceived to be more challenging in both federalized or more privatized healthcare systems which have a relative lack of control over the system – both in terms of policy and funding.

 “*We are not the one providing healthcare, we are not – technically – the ones financing the system, but in the end, we are the ones ultimately responsible for the entire system, so there is always the question: ‘what is our role and responsibility?’” *[P10].

 With regards to implementation, participants from countries with a larger proportion of private providers described having a greater reliance on encouragement and a less clearly defined role. Some participants speculated that this work is surely much easier in a single-payer system which can use centralized mandates.

####  Accountability to Commitments

 A widely discussed issue was the challenge of both being accountable to net zero commitments and meeting interim goals. Some participants discussed this issue primarily in terms of internal accountability – how to divide up the carbon budget within the health sector and allocate responsibility for cutting each chunk of emissions. Others focused on external accountability to specific targets – how to demonstrate that the health sector commitments, taken altogether, are ambitious and sincere. The development of a national strategy was seen to serve both functions, with the additional value of bringing together often already pre-existing, but piecemeal, commitments in one place.

 “*We decided to write a climate strategy as, hopefully, a yard stick to measure ourselves against and call ourselves to account in becoming way more responsible about how we protect our environment and our climate” *[P7].

 Participants also described not yet having enough clarity on what exactly net zero healthcare entails, while at the same time recognizing the power of aspirational targets to motivate staff, drive action and innovate solutions. Striking the right balance between ambition and credibility, including setting a date to reach net zero, was viewed as crucial. However, as one participant put it, whole PhD’s could be written exploring the right level of emission reductions – the focus should be on how to cut emissions without delay.

####  International Collaboration 

 The role for international collaboration was widely discussed in relation to developing strategies and inspiring partner countries to act. This was seen to help avoid needless mistakes, meet common goals, and take rapid action. Tackling Scope 3 emissions through aligning procurement expectations internationally was another area of great promise.

 “*For me the supply chain draws attention to the need for healthcare systems around the world to be working in harmony, on the same page with the same aim” *[P4].

 Participants highlighted the potential value of shared learning between high-income and low-income countries – not least, as one participant put it, given the imperative that high-income countries learn to do more with less. Alongside the potential benefits of international collaboration participants described a potential drawback: collaboration takes time. Especially for the frontrunner countries offering support to others. Given the constrained capacity of an already limited pool of people, a priority was ensuring international collaborations are, as far as possible, mutually beneficial for all country counterparts.

###  Prioritising Net Zero

 The final theme on *Prioritization* has three sub-themes: “*Conceptualizing trade-offs,”* exploring how participants framed the opportunities and challenges of pursuing net zero; “*Financing net zero,”* focusing more specifically on the anticipated financial costs; and “*Navigating trade-offs,”* piecing these two sub-themes together to explore how potential trade-offs on the pathway to net zero healthcare could and should be managed by the health sector.

####  Conceptualizing Trade-offs

 While trade-offs were considered relevant to net zero healthcare, several participants felt it important to be clear about the nature of the choices health decision-makers face. Participants wanted to distinguish between *theoretical* trade-offs and *practical* trade-offs. For example, the current use of relatively high-carbon single-use surgical equipment, as opposed to relatively low-carbon reusable equipment, to “ostensibly” minimise infection risk, was described as a misperceived trade-off.

 “*There will be people who say ‘yes’ but it has to be safe for patients. “Whatever we do has to be safe for patients” – that corollary is often put in. And it should be put in so long as its not just a wedge to kind of open the door and let the emissions out – which it could be” *[P7].

 Another participant discussed how there is a “*myth out there”* that net zero work will eat into clinical budgets, when in practice the net zero budget and the clinical budget almost never meet. Other participants described how net zero investments often save money. The *practical* trade-offs were perceived to lie more in the attention paid to the issue by policy-makers, how services are organized and the willingness to take responsibility for cutting emissions.

 Another recurring topic was the idea of “*win-wins”* or “*co-benefits”* – interventions which are both good for health and the climate. Participants reflected that there are both untapped “co-benefits,” such as telehealth to tackle urban-rural inequalities in care, but also potentially unavoidable health-climate trade-offs in the future once the relatively easy measures, such as switching from high polluting to low polluting anaesthetic gases, have been undertaken. For several participants, the relative magnitude of the potential co-benefits, as compared to the potential costs, remains marred by uncertainty.

####  Financing Net Zero

 Like all net zero targets, a central challenge described by participants was the question of what financial costs are associated with pursuing net zero healthcare. No country had fully costed the net zero programme—though some were further advanced than others—with participants explaining that they still didn’t have enough information to do so. Most of the anticipated costs were upfront capital costs on buildings and infrastructure.

 Perspectives on financing were broadly split into two categories. There were those who were primarily concerned about the immediate budgetary impacts: “*At some time you will run out of quick wins and have to do things that cost money. I’m not sure what is going to happen. There is no budget for this” *[P9].There were others who anticipated efficiency savings and viewed ‘costs’ as a misnomer, referring instead to “investments”: *“you can act on a solid 70% of your emissions profile without additional upfront capital pressure” *[P2].

 Reflections on the challenge of financing net zero also differed substantially between country income groups. An LMIC participant focused on how climate change is already having huge health impacts and they need resources immediately to help accelerate this work. By contrast, several high-income country participants described how this issue still largely receives lip service, with one stating that decision-makers will only wake up once the consequences are on their doorstep.

####  Navigating Trade-offs

 There were a range of views on the nature of the trade-offs involved in net zero healthcare. A simple cost vs. climate formulation was generally considered too narrow and out of step with the reality of health policy-making and resource allocation in healthcare. There was a desire to, as far as possible, avoid trade-offs through identifying synergies between health and climate goals.

 “*We just have to make sure that there aren’t trade offs ! I’m not sure they are necessarily naturally aligned… but we have to identify solutions that don’t compromise quality or safety in any way but also meet the goals of resilience and emissions reduction” *[P6].

 Participants also described a need to better acknowledge the *existing* trade-offs by properly accounting for climate-related harms from healthcare-related emissions. Procurement was seen to represent a good microcosm of the trade-offs at stake. Putting a value on reducing emissions (ie, a “carbon weighting”) within decision-making processes could influence decision-making – otherwise it is too easy to ignore. As one participant put it, if the clinical impact of an intervention is negligible (eg, using a high-carbon vs. a low-carbon inhaler) then patient factors need not necessarily win out.

 A related issue was the challenge of implementation. Multiple participants described how their teams were beginning to explore how to, in practice, incorporate emissions into resource allocation and procurement decisions. This was seen as an interesting development but only one piece of the puzzle.

## Discussion

 This study aimed to explore how civil servants leading the development and implementation of net zero healthcare programmes frame their responsibility to cut emissions and characterize the potential challenges and trade-offs along the way. Results from the thematic categories emerging from the interviews are summarized in the following four key findings: (1) Healthcare systems should take responsibility for their entire carbon footprint, including the harm inflicted beyond their national borders; (2) There are indications of multi-scalar health leadership—clinical, civil service, and political—helping to accelerate the net zero healthcare agenda; (3) Intersectoral collaborations necessitated by the net zero healthcare agenda could help leverage societal health improvements more broadly; and (4) Participants generally reject the notion of direct ‘trade-offs’ between efforts to reduce emissions and patient care.

 The first key finding relates to the perceived responsibility of national health systems to cut their global carbon footprint. This reflects the findings of diverse surveys and qualitative studies with healthcare workers which indicate high levels of concern about climate change, a responsibility to increase public awareness and a duty to minimise environmental impacts of healthcare.^46-48^ Clinicians are currently leading an array of work to measure and mitigate the carbon footprint of healthcare in ways which protect and improve health outcomes.^49-51^ Sherman et al argue that given the need to transform healthcare culture and practice, *“Achieving net zero emissions in healthcare will be possible only with radical and immediate engagement of the clinical community.”*^52^

 Against the backdrop of widespread engagement of the clinical community, it is particularly notable that the axiom “do no harm,” referring to the foundational principle of clinical ethics to protect patients,^53^ echoed across our interviews. This idea clearly holds rhetorical appeal – it is widely alluded to in the climate and health academic and non-academic literature, including the name ofa partner organization in the COP26 Health Programme (“Healthcare Without Harm”). As ethicist Daniel Sokol^54^ has discussed, “do no harm” is clearly a “*flawed dictum*” – more accurate would be “*Do no net harm.*” On the level of individual patients, this is what ‘do no harm’ means in practice. All healthcare interventions carry some harm, or risk of harm, which must be balanced against the potential benefits. However, where multiple people are concerned—ie, where different individuals stand to gain or lose out—it is a moral problem. In the case of carbon emissions associated with healthcare, where the benefits are accrued to individual patients while the harms, even when quantified,^55–57^ are shared globally, over time and to non-identifiable individuals,^58^ this is especially challenging for the traditional conceptualization of fair resource allocation within health systems.

 Net zero healthcare thus presents a new perspective on a longstanding problem. As long discussed by global justice theorists,^59,60^ an intimately connected global society has precluded the establishment of mechanisms to fairly govern these relations. This idea has been widely discussed in relation to healthcare as well; be it the unethical overseas procurement of surgical equipment,^61^ the “brain drain” of healthcare workers from the global south to the global north,^62^ or the inequitable distribution of vaccines during the COVID-19 pandemic.^63^ Carbon emissions further illustrate healthcare’s global impacts and the inability of national borders to delineate responsibility and fair resource allocation. Enhancing ongoing efforts to quantify and communicate the harms resulting from carbon emissions,^56,64^ including from the health sector itself,^13,65^ could support more direct accountability for the negative impacts and the responsibility to act.

 The second key finding is the role of multi-scalar leadership in driving climate action in the health sector. This clearly aligns with the rationale of the polycentric governance approach, underpinning the Paris Agreement, which posits that action across many levels of governance, rather than at the top political level alone, can inspire greater ambition and ultimately better curtail global temperature rise.^66^ However, a persistent challenge for national climate governance is the simultaneous need for both long-term strategic commitments (ie, a net zero target date) and near-term actions within the messy reality of domestic politics. The WHO *Operational Framework for Building Climate Resilient and Low-Carbon Health Systems*^67^ addresses this head on, placing transformative leadership and governance—which includes high-level political buy-in, strong governance, clear policy programmes, and synergistic intersectoral collaborations—a key pillar of its work. Of relevance for politicians and policy-makers, participants in our study set a high value on political support for net zero healthcare, describing how the COP26 Health Programme commitments helped to elevate and further legitimize their work amongst peers and staff. This may reflect the position of civil servants in the policy-making process and their need for specific goals, broad support and clear accountability mechanisms. It also highlights the value of political support as an important driver of system wide action.

 There are a growing number of case studies from the facility level^68-70^ and within clinical specialities^71,72^ showcasing actions to decarbonize healthcare. Sustaining the long-term strategic priority of net zero healthcare while avoiding piecemeal implementation is, in our view, contingent on defined targets with accountability mechanisms to monitor progress. Dedicated national net zero healthcare strategies are therefore vital, serving three main functions: first, reducing dependency upon other state and non-state actors to fulfil their climate pledges (thereby indirectly reducing healthcare’s carbon footprint), which often fade away in the face of other concerns^73,74^; second, benchmarking progress by setting out clear plans to address the bulk of emissions directly under the health sector’s control; and third, stimulating new collaborations across sectors to tackle emissions outside the health sector’s direct control, helping accelerate wider societal action.

 The third key finding is the emergence of new collaborations through net zero healthcare initiatives which could potentially improve health more broadly. This includes greater cross-sectoral collaboration which can help tackle the underlying social determinants of health, as well as international cooperation to align procurement expectations and share best practice. Bi-directional learning from countries in the global north and global south may help question well established orthodoxies: why is the carbon footprint of a cataract surgery 30 times lower in India than in the United Kingdom, at a fraction of the cost, with the same clinical outcomes?^75^ Re-framing the narrative could help turn attention to the inherent wastefulness and inefficiency of many wealthy countries’ healthcare systems and help bring the goal of ‘frugal innovation’ and low-carbon innovation into the mainstream.^76-78^

 Our final key finding is how participants conceptualise trade-offs. Although several studies frame net zero healthcare in terms of trade-offs between competing health system goals,^5,79,80^ participants were generally wary of the notion of a direct trade-off between emissions-related climate harms and patient care. This is relevant to work examining how to integrate carbon emissions into healthcare resource allocation processes.^81,82^ Several participants framed the path to net zero healthcare in terms of “investments” – emphasizing that many (so-called) “costs” provided a rapid return – or focused on “co-benefits” of climate action. Quitmann et al^34,35^ found more of a perceived conflict between mitigation and patient care than we did here – including an overriding focus to help *immediate* patients, and relatively little attention to “co-benefits” or the idea that healthcare has a special moral obligation to mitigate emissions. This may reflect differences in the setting of our study, which focused on the health policy level and so can consider the broad suite of co-benefits on a population level,^83^ rather than Quitmann et al, whose interviews with hospital administrators focused more on specific and practical mitigation measures.

 While there are indications that net zero healthcare can save money and improve health,^84,85^ there remains uncertainty as to if, and how, this can be done at pace and scale. A challenge recently discussed by Shojania^42^ is the paucity of clear-cut examples through which changes to clinical practice can reduce emissions, leading to a focus on potentially intractable problems such as reducing unnecessary care. Similarly, there remains a lack of clarity on the extent to which disease prevention can reduce the overall healthcare carbon footprint system-wide, as opposed to within individual disease pathways.^86,87^ As discussed by Sue-Chue-Lam et al,^88^ improving efficiency will not necessarily reduce emissions either, highlighting a broader argument for system transformation as opposed to changes on the margins.^89,90^

 System transformation includes change both within the system, for example, rebalancing health systems away from resource intensive hospital-based care models,^91^ and outside the healthcare system, for example, by tackling income inequality—a major driver of disease and healthcare utilization inequalities^92^—through more serious action on the social determinants of health.^93^ This alludes to a point raised by several participants that trade-offs are not simply between health and financial cost, but other factors such as political will and how services are organized; in turn, shaped by historical path dependencies which limit the space for system transformation.^94,95^ Here the WHO *Operational framework for building climate resilient and low carbon health systems* offers a useful blueprint, helping to define the characteristics of sustainable healthcare systems, identify potential trade-offs and monitor progress towards high-quality care for all in a warming world.^67^ Further empirical research characterizing the types of constraints and trade-offs policy-makers face on the path to net zero healthcare, including examples of how these have been overcome, could help integrate climate concerns into healthcare decision-making and resource allocation processes.

###  Strengths and Limitations

 Key strengths of this study are its timing—as net zero healthcare shifts from its conceptualization to implementation, and its novelty—as the first study of civil servants tasked with designing and implementing net zero healthcare initiatives. These perspectives contribute to a limited pool of empirical studies to inform both further theoretical work and policy-making. We faced several limitations. Firstly, the study focuses on high-income countries, reflecting the preliminary stage of this net zero agenda, with national commitments yet to be fully institutionalized at the country level. We faced significant challenges recruiting more participants and would ideally have included more diverse perspectives. Future research could focus on LMICs, which face distinct sets of challenges to high-income countries. Secondly, research interviews were conducted just 12-18 months following the launch of the COP26 Health Programme. As a result, participants may represent the “frontrunners” with pre-existing commitments and a history of work in this area, which could further limit the study’s generalizability. Thirdly, interviews were undertaken online and only in an individual (1:1) format. Given the timing of the interviews, at the tail end of the COVID-19 pandemic, participants were familiar with the online interview setting and talked candidly. However, they may have benefitted from focus groups to elucidate richer and potentially more nuanced perspectives on responsibilities and trade-offs. Fourth, the interviews were undertaken, transcribed, and primarily analyzed by the lead author, as part of their PhD project. We took steps throughout the research process to minimize individual bias affecting the study’s validity,^96^ including independently reading the transcripts and identifying preliminary themes, and having regular discussions at each stage of the research process, from the study conceptualization to the interview guide design, data analysis and write up.

## Conclusion

 The shift towards more sustainable, low-carbon healthcare systems presents new ethical challenges and potential trade-offs for health systems which have traditionally overlooked the climate impacts of healthcare delivery. This study offers a snapshot of the net zero healthcare agenda in its early phases, primarily in high-income countries. Our study highlights both the sincerity of country commitments to delivering net zero healthcare systems and uncertainties on how to get there. Notably, participants generally rejected the simplistic framing of health vs. climate trade-offs, favouring the language of “investments” over “costs.” They also highlight the moral responsibility of health systems to tackle their carbon footprint and the potential to inspire greater action in other sectors. For the growing number of countries working to develop net zero healthcare strategies, these findings provide an insight into how country counterparts are conceptualizing and working towards net zero healthcare. To support this work, future research should aim to develop a richer empirical understanding of the constraints and trade-offs civil servants and policy-makers face, with specific attention to the challenges and opportunities the net zero healthcare agenda presents in LMICs.

## Acknowledgements

 This project was conducted with the support of the Takemi Program in International Health at the Harvard T.H. Chan School of Public Health.

## Ethical issues

 The study has received ethical approval from the Norwegian Centre for Research Data (Reference number: 367779). No patient sensitive or personal data were collected. All participants were provided with material explaining the aims, data management and participation requirements in advance of the interview and provided written consent to take part in the study.

## Conflicts of interest

 AB and OFN are members of the Lancet Commission on Sustainable Healthcare. KB has no conflicts of interest to declare.

## Availability of data and materials

 Given the relatively small number of potential participants, demographic data on participants are not provided and to the minimal extent necessary, quotes and text have edited to remove any identifiable information.

## Supplementary files

Supplementary file 1. Interview Guide.
