# Supplementary material for: Ambition With Uncertainty: Exploring Policy-Makers’ Perspectives on Pathways to Net Zero Healthcare
Source: Int J Health Policy Manag. 2025 Jan 15;14:8440. doi: 10.34172/ijhpm.8440 (PMC11845860; doi:10.34172/ijhpm.8440)
Supplement: Supplementary file 1 — Interview Guide. [file ijhpm-14-8440-s001.pdf]

Article title: Ambition With Uncertainty: Exploring Policy-Makers' Perspectives on Pathways to Net Zero Healthcare

Journal name: International Journal of Health Policy and Management (IJHPM)

Authors' information: Anand Bhopal[1,2,3]*, Kristine Bærøe[4], Ole F. Norheim[1,5]

[1]Bergen Centre for Ethics and Priority Setting, Department of Global Public Health and Primary Care, Faculty of Medicine, University of Bergen, Bergen, Norway.

[2]Centre for Energy and Climate Transformation (CET), University of Bergen, Bergen, Norway.

[3]Takemi Program in International Health, Department of Global Health and Population, Harvard T.H. Chan School of Public Health, Harvard University, Boston, MA, USA.

[4]Department of Global Public Health and Primary Care, Faculty of Medicine, University of Bergen, Bergen, Norway.

[5]Department of Global Health and Population, Harvard T.H. Chan School of Public Health, Harvard University, Boston, MA, USA.

***Correspondence to:** Anand Bhopal; Email: anand.bhopal@uib.no

Citation: Bhopal A, Bærøe K, Norheim OF. Ambition with uncertainty: exploring policy-makers' perspectives on pathways to net zero healthcare. Int J Health Policy Manag. 2025;14:8440. doi:10.34172/ijhpm.8440

Supplementary file 1. Interview Guide

1) Setting the scene

a. Please could you please tell me about your role within the health system/ministry/directorate?

b. Can you give me some background on healthcare decarbonisation in xxxx?

c. What do you think are the reasons xxxx signed up to the COP26 health programme?

2) Responsibility for healthcare carbon emissions

a. In your view, what is the responsibility of your healthcare system to cut emissions?

b. In your view, is healthcare different as compared to other sectors – should it have more or less or the same amount of time to reach net zero?

3) Setting priorities

a. Could you share with me your impression of how to reach net zero emissions in your healthcare system?

b. Could you describe some of the challenges you face in delivering this agenda?

c. In general, how do you think policy-makers in your country should balance healthcare decarbonisation with other health sector priorities?

d. Are there any trade-offs between healthcare provision and the climate mitigation targets?

***4)* International perspective**

a) Could you describe how you think responsibility for cutting health sector emissions should be shared globally?

b) In your view, does the healthcare sector have responsibility to reduce Scope 3 emissions?

***5)* Closing:**

a. Is there anything more you would like to add?
